# A novel approach to the follow up of children with otitis media with effusion: wideband absorbance findings

**DOI:** 10.1007/s00405-025-09355-3

**Published:** 2025-04-12

**Authors:** Melis Keskin Yıldız, Gamze Atay, Esra Kutsal Mergen, Songül Aksoy, Bilgehan Böke

**Affiliations:** 1 Department of Audiology, Faculty of Health Sciences, Gazi University, Ankara, Turkey; 2 Department of Otolaryngology, Faculty of Medicine, Hacettepe University, Ankara, Turkey; 3 Department of Biostatistics, Faculty of Medicine, Hacettepe University, Ankara, Turkey; 4 Department of Audiology, Faculty of Health Sciences, Lokman Hekim University, Ankara, Turkey; 5 Vocational School of Health Services, Hacettepe University, Ankara, Turkey

**Keywords:** Absorbance, Otitis media with effusion, Wideband absorbance, Pneumatic otoscopy, Wideband tympanometry

## Abstract

**Purpose:**

Otitis media with effusion (OME) is one of the most common childhood diseases. It is recommended to use tympanometry in addition to otoscopy and/or pneumatic otoscopy for the diagnosis and follow-up of OME. Clinitians are using Wideband absorbance (WBA), which is one of the methods of evaluating the middle ear in the diagnosis of OME, more widely.

**Methods:**

The relationship between the changes in the examination findings obtained by otoscopy and pneumatic otoscopy and the findings of WBA in the monthly follow-ups performed during the three-month period of children diagnosed with OME, was examined. In the study, 48 ears of 26 individuals aged 24–71 month who were diagnosed with OME were evaluated. Otoscopy, pneumatic otoscopy, 226 Hz tympanometry and WBA measurements were performed at the initial, first, second and third month examinations. The relationship between the difference between consecutive measurements was examined because it was thought that consecutive measurements might be more significant in the relationship between measurements. The Spearman test was used to determine whether there was a relationship between WBA and otoscopic and pneumatic otoscopic examination results. For assessing parameters where there was a significant difference between the groups compared, the variables were assessed using Kruskal-Wallis analysis of variance. The Dunn test was used for post hoc testing.

**Results:**

The change observed with both otoscopy and pneumatic otoscopy between the initial and first month control could not be adequately determined by 226 Hz tympanometry, but the change in the amount absorbance at 2520 Hz and 3175 Hz was moderately correlated with the examination findings. However, it was observed that the change between the first and the second follow-up examinations was significant at the frequency range of 226–630 Hz in WBA and the change between the second and third months was significant at the frequency range of 226–4000 Hz in WBA.

**Conclusions:**

The findings obtained in the study show that, 226 Hz tympanometry may be insufficient to reflect the change in examination findings while WBA can provide more detailed information to support the examination findings during the follow-up period as well as the diagnosis of OME.

## Introduction

Otitis Media with Effusion (OME) is defined as the presence of fluid in the middle ear without signs and symptoms of acute infection. It very common causes of hearing loss in childhood [1, 2]. Otoscopic examination is the most basic component of physical examination in the diagnosis of OME. It has become important to current clinical practice guidelines in many respects, including its prevalence, difficulty in diagnosis, potential impact on child development, and practice changes in treatment [3]. Although pneumatic otoscopic examination is recommended when diagnosing OME in the clinical practice guide, it is emphasized that it has varying degrees of validity and reliability in routine clinical applications [3]. Current clinical guidelines recommend a 3-month observation period with clinical tests [3]. The 3-month follow-up period is to prevent unnecessary surgeries. According to the clinician’s preference, this period consists of otoscopic methods or intermittent controls in which tympanometry test is performed in addition to otoscopic methods [3]. Tympanometric evaluation provides useful information in interpreting otoscopic examination findings. Conventional (226 Hz) tympanometry is among the routine audiological test battery which is used to evaluate the middle ear sound conduction system. However, 226-Hz tympanometry may be insufficient to detect slight changes in middle ear mechanics [4]. Since the presence of fluid in the middle ear cannot be determined definitively without myringotomy, reliable findings are needed to guide clinicians in the diagnosis and follow-up process [3, 5, 6]. Tympanometry and/or pneumatic otoscopy are insufficient to provide information about effusion in some cases and 226 Hz tympanometry does not have a consistent effect in determining the effusion volüme. Wide Band Tympanometry (WBT), which evaluates the acoustic response properties of the middle ear in a wide frequency range (226–8000 Hz), is a powerful and sensitive indicator of middle ear effusion volume in children with otitis media with effusion. Unlike conventional tympanometry, absorption values can also be obtained at ambient pressure. Wide band tympanometry provides a multi-dimensional assessment of middle ear function, with the absorption curve plotted as a joint function of frequency and pressure. This wide-band assessment of middle ear function provides detailed information about the status of the middle ear, which can significantly facilitate diagnosis. therefore, studies conducted with WBT and Wide Band Absorbance (WBA) in the diagnosis and follow-up of OME have been emphasized recently [an^4^, ^7^–^12^].

It has been emphasized in many studies that WBA is more sensitive in detecting middle ear effusion compared to 226-Hz tympanometry [5, 13]. There are few numbers of studies evaluating the relationship between examination findings and the amount of absorbance [5, 14, 15]. In addition, no study has been found to evaluate the relationship between the changes in membrane findings determined by otoscopy and pneumatic otoscopy with periodic controls and WBA. In this regard, our study is especially important in terms of supporting otoscopy/pneumatic otoscopy findings and guiding the clinician.

## Materials and methods

This study was conducted in a university hospital. The local ethical committee approved this study (date: 24.05.2018, number: KA-17162 2018/08–28). A total of 30 volunteers who diagnosed with OME were followed up with monthly controls for 3 months from the diagnosis. Otoscopic examination, pneumatic otoscopic examination, 226 Hz tympanometry and wideband tympanometry tests were performed on individuals at the initial examination and controls. However, 2 individuals (2 ears) in the initial evaluation, 1 individual (2 ears) in the second control, and 1 individual (1 ear) in the third control could not cooperate with otoscopy and/or pneumatic otoscopy, and therefore no examination findings could be obtained. Therefore, the study was completed with 26 individuals (48 ears).

### Otoscopic examinations

Ear examinations of individuals were performed by a single otolaryngologist, who experienced with a ten year tenure in the feld, to avoid observational differences, and the physician was blinded to wideband absorbance results and conventional tympanometry results to avoid biases. In addition to demographic data, active complaints and medical background inquiries were made. The presence of chronic disease (especially leading to ciliary dysfunction or immunodeficiency), drug use, ear surgery history, especially ventilation tube application, tonsillectomy-adenoidectomy history, and cleft palate-lip surgery history were questioned in the medical history. Children with any of these were not included in the study. OME was detected during the examination when individuals presented with complaints such as frequent URTI, sleeping with open mouth-snoring, ear pain, hearing loss, speech delay and lack of attention. OME was diagnosed in accordance with the definition in the clinical practice guideline [3].

Routine otoscopy and pneumatic otoscopy, 226 Hz tympanometry and wideband tympanometry evaluations were performed on the individuals who were accepted to participate in the study by their families, at the initial examination, and at the first, second and third months when they came for control.

In line with the aim of the study, it was based on the method of the study of Ellison et al. [5] in which they examined the findings of WBT in children with OME. In pneumatic otoscopy, the physician categorized tympanic membrane mobility as 1–5; “1 normal”, “2 slightly stiff”, “3 moderately stiff”, “4 very stiff” and “5 no movement”. According to the condition of the membrane in otoscopy findings, one or more of the normal, slightly dull, dull, vascular and retracted appearances were noted. It was classified as “1 normal”, “2 slightly dull appearance”, “3 dull appearance”, “dull and vascular appearance 4”, “retracted appearance 5”.

### Tympanometric measurements

On the same day after the otoscopic examinations of the patients, wideband tympanometry and 226 Hz conventional tympanometry measurements were performed in a single session. Interacoustics Titan version 3.1 was used for WBT measurements.

WBT were performed at frequencies between 226 Hz and 8000 Hz using a suitable probe tip placed external to the ear canal. The data obtained from 226–297,3–385,55–500–629,96–793,7–1000 − 1259,92–1587,4–2000 − 2519,84–3174,8–4000 − 5039,68–6349,6–8000 Hz in 1/3 octave frequencies were analysed for ambient and peak pressure absorbance evaluations.

As a result of the 226 Hz tympanometry measurement, the results were classified as type A, type B and type C [16]. Those with a negative pressure ≤-100 daPa at normal compliance were classified as type C tympanograms, and those with a positive pressure of more than − 100 daPa were classified as type A tympanograms. Those with an abnormally low admittance with no discernible peak were classified as type B. After the measurement had finished, the WBA data obtained were transferred to the target folder determined by a software prepared according to the device, and the data was transferred to the excel file prepared by the device company used.

### Statistical analysis

SPSS for Windows version 23.0 were used for the statistical analysis. Whether the data showed normal distribution or not was determined by Kolmogorov-Smirnov test. Statistics of normally distributed data are shown with mean and standard deviation values, and statistics of non-normally distributed data are shown with median values.The significance level of *p* < 0.05 was used in all statistical analyses. It was thought that consecutive measurements could be more significant in the relationship between measurements, therefore, the relationship between the difference between consecutive measurements was examined. In order to determine whether there is a relationship between the WBA and otoscopic and pneumatic otoscopic examination findings, the “Spearman Test” was applied. The relationship between otoscopic and pneumatic otoscopic examination findings and absorbance change at 1/3 octave frequencies in WBA for both peak pressure and ambient pressure measurements (initial examination– first control, First control– second control, Second control– third control) were analyzed.

In the assessment parameters where there was a significant difference between the groups compared, the variables were assessed using Kruskal-Wallis analysis of variance, as the variables did not show a normal distribution. The alpha level of significance was taken as 0.05 and the cases where the (p) value was below 0.05 (*p* < 0.05) were considered statistically significant. In cases where there was a significant difference between the groups (*p* < 0.05), the groups were compared in pairs using post-hoc tests to determine which group was responsible for the difference. The Dunn test was used to determine the post hoc tests.

## Results

A total of 48 ears belonging to 26 individuals aged between 24 and 71 month (M:13,F:13, mean age: 52,96 ± 14,641 (month)) were evaluated. Our primary aim in the study is to examine the relationship between examination findings and WBA results during OME follow-up.

The findings of otoscopy and pneumatic otoscopy parameters were presented in Table 1a and 1b.

**Table 1a:** Otoscopic examination findings

	Initial examination		First control		Second control		Third control	
	*n*	%	*n*	%	*n*	%	*n*	%
Normal	0	0.00	7	14,6	5	10,4	14	29,2
Slightly dull	5	10,4	4	8,3	7	14,6	8	16,7
Dull	13	27,1	13	27,1	11	22,9	8	16,7
Dull and vascular	22	45,8	17	35,4	16	33,3	13	27,1
+ Retracted	8	16,7	7	14,6	9	18,8	5	10,4
Total	48	100,00	48	100,00	48	100,00	48	100,00

**Table 1b:** Pneumatic otoscopic examination findings

	Initial examination		First control		Second control		Third control	
	*n*	%	*n*	%	*n*	%	*n*	%
Normal	0	0,00	6	12,5	5	10,4	13	27,1
Slightly stiff	7	14,6	5	10,4	9	18,8	6	12,5
Moderately stiff	10	20,8	13	27,1	7	14,6	8	16,7
Very stiff	17	35,4	14	29,2	14	29,2	7	14,6
No movement	14	29,2	10	20,8	13	27,1	14	29,2
Total	48	100,00	48	100,00	48	100,00	48	100,00

Table 1a shows the distribution of findings obtained in the otoscopic examination. According to the otoscopic examination findings; in the initial evaluation, slightly dull in 5 (%10.4) ears, dull in 13 (%27.1) ears, dull-vascular in 22 (%45.8) ears, and retracted membrane findings were obtained in 8 (%16.7) ears. In the first control, normal in 7 (%14.6) ears, slightly dull in 4 (%8.3) ears, dull in 13 (%27.1) ears, dull-vascular in 17 (%35.4) ears, and retracted membrane findings were obtained in 7 (%14.6) ears. In the second control, normal in 5 (%10.4) ears, slightly dull in 7 (%14.6) ears, dull in 11 (%22.9) ears, dull-vascular in 16 (%33.3) ears, and retracted membrane findings were obtained in 9 (%18.8) ears. At the third control, normal findings were found in 14 (29.2%) ears, slightly dull in 8 (16.7%) ears, dull in 8 (16.7%) ears, dull-vascular in 13 (27.1%) ears, and retracted membrane findings were found in 5 (10.4%) ears.

Table 1b shows the distribution of findings obtained in the initial evaluation and controls in pneumatic otoscopic examination. According to the pneumatic otoscopic examination findings; In the initial evaluation, slightly stiff in 7 (%14.6) ears, moderately stiff in 10 (%20.8) ears, very stiff in 17 (%35.4) ears, and no-movement membrane findings in 14 (%29.2) ears were obtained. In the first control, normal in 6 (%12.5) ears, slightly stiff in 5 (%10.4) ears, moderately stiff in 13 (%27.1) ears, very stiff in 14 (%29.2) ears, and no movement membrane findings in 10 (%20.8) ears were obtained. In the second control, normal findings were obtained in 5 (10.4%) ears, slightly stiff in 9 (18.8%) ears, moderately stiff in 7 (14.6%) ears, very stiff in 14 (29.2%) ears, and no movement membrane findings were obtained in 13 (27.1%) ears. In the third control, normal findings were obtained in 13 (27.1%) ears, slightly stiff in 6 (12.5%) ears, moderately stiff in 8 (16.7%) ears, very stiff in 7 (14.6%) ears, and no movement membrane findings were obtained in 14 (29.2%) ears.

When the relationship between otoscopic and pneumatic otoscopic examination findings is examined, it is seen that the positive correlation value between otoscopic and pneumatic otoscopic examination is high (strong) in the initial examination (*r* = 0,710), first (*r* = 0,712), second (*r* = 0,817) and third (*r* = 0,842) control. However, it was observed that the amount of association increased gradually at each control (*p* < 0,05).

The 226 Hz tympanometry results of the individuals measured at the initial examination and controls are presented in Table 2.

**Table 2:** 226 Hz tympanometry findings

Type of Tympanogram	Initial examination		First control		Second control		Third control	
	*N*	%	*N*	%	*N*	%	*N*	%
Type A	1	2,1	5	10,4	4	8,4	19	39,6
Type B	34	70,8	28	58,3	22	45,8	10	20,8
Type C	13	27,1	15	31,3	22	45,8	19	39,6
Total	48	100,0	48	100,0	48	100,0	48	100,0

When the relationship between otoscopic examination findings and 226 Hz tympanometry findings are examined: there is a significant relationship in the initial examination (*r* = 0,288), in the second (*r* = 0,572) and third (*r* = 0,605) controls, but not in the first control (*p* < 0.05).

When 226 Hz tympanometry and otoscopic examination findings were analysed by Kruskal-Wallis Analysis of Variance, it was found that there was no significant difference in otoscopic examination findings and 226 Hz tympanometry measurements made at the initial evaluation (*p* > 0.05). In other words, there was no statistically significant difference between the distributions of otoscopic examination results in ears with type A, type B and type C tympanograms. However, there was a statistically significant difference for the evaluations performed at the first, second and third month controls (*p* < 0.05). In the measurements where a significant difference was obtained, a pairwise comparison was made between the subgroups to determine which group or groups the difference originated from, and when the results were analysed, in terms of otoscopy findings:

For the first month control, it was observed that there was a difference between the groups with type A - type C tympanograms and between the groups with type A - type B tympanograms, but there was no difference between the groups with type C - type B tympanograms in terms of examination findings.

For the second month control, the difference was observed between the groups with type A - type B tympanograms and between the groups with type C - type B tympanograms, but there was no difference between the groups with type A - type C tympanograms in terms of examination findings.

For the third month control, the difference was observed between the groups with type A - type B tympanograms, but there was no difference between the groups with type C - type B tympanograms and the groups with type A - type C tympanograms in terms of examination findings.

When the relationship between pneumatic otoscopic examination findings and 226 Hz tympanometry findings are examined: there is a significant relationship in the initial examination (*r* = 0,293), in the first control (*r* = 0,481), in the second (*r* = 0,586) and third (*r* = 0,708) control (*p* < 0.05).

When 226 Hz tympanometry and pneumatic otoscopic examination results were analysed by Kruskal-Wallis Analysis of Variance, it was observed that there was no significant difference in pneumatic otoscopic examination findings and 226 Hz tympanometry measurements at initial evaluation (*p* > 0.05). In other words, there was no statistically significant difference between the distributions of pneumatic otoscopic examination findings in ears with type A, type B and type C tympanograms. However, there was a statistically significant difference for the measurements made at the first, second and third month controls (*p* < 0.05). In the measurements where a significant difference was obtained, a pairwise comparison was made between subgroups to determine which group or groups the difference originated from, and when the results were examined, in terms of pneumatic otoscopy findings:

For the first month control, it was observed that there was a difference between the groups with type A - type C tympanograms and between the groups with type A - type B tympanograms, but there was no difference between the groups with type C - type B tympanograms in terms of examination findings.

For the second month control measurements, the difference was observed between the groups with type A - type B tympanograms and between the groups with type C - type B tympanograms, but there was no difference between the groups with type A - type C tympanograms in terms of examination findings.

For the third month control measurements, the difference was observed between the groups with type A - type C tympanograms and between the groups with type A - type B tympanograms, but there was no difference between the groups with type C - type B tympanograms in terms of examination findings.

The relationship between otoscopic and pneumatic otoscopic examination findings and the change in absorbance at 1/3 octave frequencies in WBA was examined for both peak and ambient pressure measurements by taking the differences for each variable in the initial assessment - first control, first control - second control, second control - third control measurements using the Spearman correlation coefficient. As a result of the analysis, only the frequencies that were significant in the WBA are shown in the Table 3.

**Table 3:** The relationship between otoscopic/pneumatic otoscopic examination findings and wideband tympanometry

		Initial examination– 1st control				1st control- 2nd control				2nd control- 3rd. control						
		Otoscopy		Pneumatic Otoscopy		Otoscopy		Pneumatic Otoscopy		Otoscopy			Pneumatic Otoscopy			
		Peak Pressure	Ambient Pressure	Peak Pressure	Ambient Pressure	Peak Pressure	Ambient Pressure	Peak Pressure	Ambient Pressure		Peak Pressure	Ambient Pressure		Peak Pressure	Ambient Pressure	
226 Hz	corelation coefficient		-0,296			-0,334	-0,39	-,381	-,412		-0,483	-,557		-,587	-,621	
	p		0,041			0,02	0,006	0,007	,004		0,001	,000		,000	,000	
297,3 Hz	corelation coefficient					-0,333	-0,371	-,313	-,379		-,473	-,551		-,522	-,606	
	p					0,021	0,009	,030	,008		,001	,000		,000	,000	
385,55 Hz	corelation coefficient					-0,361	-0,429	-,427	-,525		-,482	-,578		-,567	-,628	
	p					0,012	0,002	,003	,000		,001	,000		,000	,000	
500 Hz	corelation coefficient				-,310	-0,333	-0,431	-,426	-,548		-,433	-,567		-,593	-,660	
	p				,032	0,021	0,002	,003	,000		,002	,000		,000	,000	
629,96 Hz	corelation coefficient				-,330	-0,304	-0,39	-,348	-,515		-,452	-,589		-,573	-,685	
	p				,022	0,036	0,006	,015	,000		,001	,000		,000	,000	
793,7 Hz	corelation coefficient				-,324		-0,345		-,477		-,504	-,615		-,511	-,714	
	p				,025		0,016		,001		,000	,000		,000	,000	
1000 Hz	corelation coefficient				-,322		-0,362		-,475		-,543	-,676		-,496	-,718	
	p				,026		0,011		,001		,000	,000		,000	,000	
1259,92 Hz	corelation coefficient						-0,309		-,425		-,561	-,710		-,504	-,732	
	p						0,032		,003		,000	,000		,000	,000	
1587,4 Hz	corelation coefficient								-,367		-,510	-,674		-,390	-,689	
	p								,010		,000	,000		,006	,000	
2000 Hz	corelation coefficient										-,483	-,525		-,336	-,579	
	p										,001	,000		,020	,000	
2519,84 Hz	corelation coefficient	-0,379	-0,359	-,363					-,362		-,387	-,496		-,310	-,521	
	p	0,008	0,012	,011					0,11		,007	,000		,032	,000	
3174,8 Hz	corelation coefficient	-0,366	-0,338	-,332							-,401	-,503		-,374	-,489	
	p	0,01	0,019	,021							,005	,000		,009	,000	
4000 Hz	corelation coefficient	-0,285	-0,294			-0,364	-0,295				-,357	-,419		-,289	-,402	
	p	0,05	0,043			0,011	0,042				,013	,003		,046	,005	

### Relationship between wideband absorbance and otoscopic examination findings

According to the correlation coefficients in frequencies where absorbance change was found significant at peak pressure and ambient pressure for the initial evaluation and first month control, the most significant negative relationship was found at 2520 Hz both at peak pressure and ambient pressure. At peak pressure; a moderate level relationship was observed at 2520 Hz, 3175 Hz and at 4000 Hz low level relationship was observed. At ambient pressure, a moderate level relationship was observed at 2520 Hz and 3175 Hz, and a low level relationship was observed at 4000 Hz and 226 Hz. Common frequencies that were significant at both peak pressure and ambient pressure were 2520 Hz, 3175 Hz and 4000 Hz (Table 3).

For the first and second month control, frequencies with significant absorbance changes are presented in Table 3. At peak pressure, a moderate relationship was found at 386 Hz, 226 Hz, 297 Hz, 500 Hz, 630 and 4000 Hz. At ambient pressure, a moderate relationship was found at 500 Hz, 386 Hz, 630 Hz, 226 Hz, 297 Hz, 1000 Hz, 794 Hz and 1260 Hz, and a low relationship was found at 4000 Hz. Common frequencies that were significant at both ambient pressure and peak pressure were found to be 226 Hz, 297 Hz, 386 Hz, 500 Hz and 630 Hz.

For the second and third month control, according to the correlation coefficients for the absorbance change at 1/3 octave frequencies at peak pressure and ambient pressure, the most significant negative relationship was found at 1260 Hz at peak and ambient pressure. At peak pressure, a moderate relationship was found at 1260 Hz, 1000 Hz, 1587 Hz, 794 Hz, 2000 Hz, 226 Hz, 386 Hz, 297 Hz, 630 Hz, 500 Hz, 3175 Hz, 2520 Hz, 4000 Hz. At ambient pressure; It was found to be highly correlated at 1260 Hz, moderately correlated at 1587 Hz, 1000 Hz, 794 Hz, 630 Hz, 386 Hz, 500 Hz, 226 Hz, 297 Hz, 2000 Hz, 3175 Hz, 2520 Hz and 4000 Hz. All frequencies that are significant at both ambient and peak pressures are common, but correlation coefficients differ.

### Relationship between wideband absorbance and pneumatic otoscopic examination findings

For the initial evaluation and first month control, the absorbance change at peak pressure and ambient pressure was found to be significant. According to the correlation coefficients, the most significant negative relationship was found at 2520 Hz at peak pressure, and a moderate relationship was found at 2520 Hz and 3175 Hz at peak pressure. At ambient pressure, a moderate relationship was found at 630 Hz, 794 Hz, 1000 Hz, 500 Hz.

For the first and second month controls, the most significant correlation coefficients for the frequencies at peak pressure and ambient pressure was found to be significant were found at 386 Hz at peak pressure and 500 Hz at ambient pressure. A moderate correlation was found at 386 Hz, 500 Hz, 226 Hz, 630 Hz, 297 Hz at peak pressure. A moderate correlation was found at 500 Hz, 386 Hz, 630 Hz, 794 Hz, 1000 Hz, 1260 Hz, 226 Hz, 297 Hz, 1587 Hz, 2520 Hz at ambient pressure. The common frequencies that were significant at both ambient pressure and peak pressure were found to be 386 Hz, 500 Hz, 297 Hz, 226 Hz, 630 Hz.

For the second and third month follow-up, the most significant negative relationship was found at 500 Hz at peak pressure and 1260 Hz at ambient pressure according to the correlation coefficients for the frequencies where the absorbance change at 1/3 octave frequencies at peak pressure and ambient pressure was found to be significant. A moderate relationship was found at 500 Hz, 226 Hz, 630 Hz, 386 Hz, 297 Hz, 794 Hz, 1260 Hz, 1000 Hz, 1587 Hz, 3175 Hz, 2000 Hz, 2520 Hz at peak pressure, and a low level relationship was found at 4000 Hz. At ambient pressure, it was found to be highly correlated at 1260 Hz, 1000 Hz, 794 Hz, and moderately correlated at 1587 Hz, 630 Hz, 500 Hz, 386 Hz, 226 Hz, 297 Hz, 2000 Hz, 2520 Hz, 3175 Hz and 4000 Hz. All frequencies that are significant at both ambient pressure and peak pressure are common, but correlation coefficients differ.

## Discussion

Diagnosis and treatment are still controversial due to the variability in clinical manifestations of OME. Otoscopic examination has a very important place in diagnosis. Otoscopic examination has been performed for many years as a way of obtaining information about the eardrum and middle ear. However, sometimes this examination causes children to cry with fear, which increases blood supply to the tympanic membrane, making diagnosis difficult. Although otoscopy alone has good sensitivity, its specificity is low [17–19].

Among the many diagnostic methods used for OME, pneumatic otoscopy is known for best assess the presence of middle ear effusion. Recent clinical practice guidelines have recommended the use of pneumatic otoscopy as the primary diagnostic method for OME [3, 6, 20]. However, many clinicians rely only on information obtained by otoscopy without using a pneumatic otoscopy [21]. Even for the most experienced ENT specialist, the diagnosis may be imprecise or difficult to obtain in some cases. Although pneumatic otoscopy has high sensitivity and specificity rates for the diagnosis of OME, its widespread use remains a target, and its routine and accurate application in daily practice remains a major problem [8]. The results of our study show that the amount of correlation (concordance) between otoscopy and pneumatic otoscopy was high (strong) in the initial examination, first, second and third control. In addition, the amount of correlation increases with each control. The statistically high level of correlation between otoscopy and pneumatic otoscopy indicates the consistency of the ratings made by the same ENT physician.

The low level of correlation between otoscopic examination and 226 Hz tympanometry findings at the initial examination and the lack of a significant correlation at the first month follow-up indicates that 226 Hz tympanometry has limitations in detecting small differences in the eardrum during OME follow-up. This finding was found to be compatible with a study [22].

In parallel with the increase in the number of healed ears in the second and third months, it was thought that a moderate relationship was obtained, and thus the increase in the degree of the relationship obtained in the third month was also related to this improvement. Similarly, in pneumatic otoscopy, the strongest correlation was observed in the third month, when improvements were in the majority. In the light of these findings, it is possible to conclude that 226 Hz tympanometry is more reliable in normal or near-normal eardrum findings. Gimsing and Bergholtz [22] reported that the agreement between pneumatic otoscopy and 226 Hz tympanometry findings was higher in normal ears than in abnormal ears. Kemaloglu et al. [23] reported that, tympanometry alone makes it more difficult to find ears without effusion between the ears that have been diagnosed clinically and otoscopically as otitis media than to find normal ears. There are other studies showing that 226 Hz tympanometry cannot reliably distinguish between normal and pathological ears [18, 19, 24–26].

In our study, the change in WBA values measured when individuals followed up with the diagnosis of OME for 3 months came to the control with 1 month intervals and the change between otoscopy and pneumatic otoscopy findings on the same day were examined. A negative correlation was found between the change in both pneumatic otoscopy and otoscopy findings and absorbance findings in all of the initial examination-first month, first month-second month, second month-third month controls. In other words, absorbance decreases in all frequencies with a significant difference in ears with more intense effusion compared to otoscopic examination findings, while absorbance increases in ears where effusion regresses due to improvement according to examination findings. In many studies, it has been reported that higher reflectance and lower absorbance are obtained in ears with OME findings [14, 27, 28]. Ellison et al. [5] graded the TM movements of the individuals in the control group from 1 to 5 by pneumatic otoscopy and examined the change in absorbance between the groups. They reported that as the TM hardness increased, the absorbance decreased. Merchant et al. reported, As the volume of the effusion increases, the absorbance systematically decreases. This reduction is present at most frequencies but is greatest in the frequency range 1 to 5 kHz [11].

There are studies in the literature evaluating absorbance findings at peak pressure and ambient pressure. Keefe et al. [10] performed 226 Hz tympanometry and WBA tests at both ambient pressure and TTB in children aged 3–8 years with conductive hearing loss due to EOM and in a control group with normal hearing. No significant difference was found between the values ​​measured at ambient pressure and peak pressure, and they reported that both absorbance tests were more successful than traditional tympanometry in correctly detecting the presence of conductive hearing loss in children. Aithal et al. [28] divided the ears with EOM into two groups according to fluid density during surgery, and in their study, they found no difference between ears with low and high fluid density at ambient pressure as a result of WBA measurement, while a difference was found between the two groups at peak pressure. Based on this result, they reported that fluid density could be distinguished by peak pressure measurements. Other studies in the literature also stated that absorbance measurements at peak pressure were more sensitive than those at ambient pressure [29]. Therefore, it is recommended that absorbance measurements be made at peak pressure [30–32].

İt is seen that most frequencies found to be related to peak pressure in WBA with otoscopic examination findings are also related to ambient pressure. Although it is seen that frequencies found to be related to peak pressure in WBA with pneumatic otoscopic examination findings are common with frequencies found to be related to ambient pressure for first-second month and second-third month changes, it is seen that more frequencies are significant in ambient pressure measurements for first-second month changes for both otoscopy and pneumatic otoscopy. In the evaluation of the relationship between otoscopy and pneumatic otoscopy-WBA, it is seen that the frequencies related to the peak pressure are also common to the ambient pressure in all measurements with a few exceptions.Therefore, comments are made considering the frequencies common to peak pressure for otoscopic and pneumatic otoscopic examination.

In our study, the common frequencies reflecting the change in otoscopy and pneumatic otoscopy between the initial examination and the first month in peak pressure and ambient pressure were found as 2520 Hz and 3175 Hz. Ellison et al. [5] reported that 1.5–3 kHz at the ambient pressure was the most sensitive frequency range in terms of OME between the group with otitis media with effusion and the control group. Taiji and Kazanski [13] found that 19 patients with OME and 8 without OME whose 226 Hz tympanometry result was type B, had lower WBA values between 2 and 4 kHz in the group with OME, compared to the group without OME. Hunter et al. [27] reported that in the presence of otitis media with effusion, there was a significant difference in the reflectance and absorbance properties of the middle ear in the frequency range of 1000–4000 Hz. Şahin et al. [33] reported that especially the high absorbance value of 1000 Hz and 2000 Hz in the follow-up of otitis media with effusion indicates that the prognosis will be good. The frequencies reflecting the change in the initial examination and the first month are quite compatible with the frequency ranges specified in the literature. In line with the findings of our study, it can be interpreted that although the effusion is still intense, the change between the initial examination and the first month which couldnt be seen on 226 Hz, can be seen with WBA. In our study, when the frequency range showing the change between the first month and the second month was examined, it was observed that the frequencies reflecting the change in both otoscopy and pneumatic otoscopy findings at peak pressure shifted from 2520 to 3175 Hz to the frequencies between 226 Hz and 630 Hz. Based on the knowledge that the presence of fluid in the middle ear cavity in the presence of negative pressure and effusion will increase the mass and stiffness of the middle ear system; it is thought that this change is caused by the gradual decrease in the mass effect in the middle ear as the effusion regresses and the negative pressure decreases. In our study, it is seen that it is possible to observe the change in absorbance between the second and third months in the frequency range from 226 Hz to 4000 Hz, both at peak pressure and at ambient pressure, without being limited only to low frequencies. Margolis et al. [34] reported that the reflectance patterns obtained with different ear canal pressures were greatly different. Voss et al. [35] reported that the increase in middle ear cavity volume systematically decreased reflectance at frequencies below 2000 Hz. Beers et al. [36] reported that as middle ear pressure changes from mildly negative to OME, reflectance consistently increases over a wider frequency range.

While the most important frequencies reflecting the change between the initial examination and the first month control for otoscopy and pneumatic otoscopy are 2520 Hz and 3175 Hz, the change in absorbance at these frequencies does not become important in terms of the change between the first and second month. The absorbance change at frequencies between 226 and 630 Hz becomes important for the change between first and second control. This finding are very important in terms of OME prognosis because studies on WBT and OME in many studies mostly emphasize that the important frequency range for OME is between 1 and 4 kHz [5, 13, 27].

The most important advantage in our study is that the same ears can be evaluated by controls and the change in otoscopic examination findings can be monitored step by step with frequency-specific absorbance change in WBA. It was thought that the different results in terms of absorbance (or reflectance) findings were due to the fact that the individuals included in the studies were not known at what stage of OME, and different methods/criteria(s) were used in the studies to diagnose OME.

### Limitations of the study

The present study has a few limitations. First, the current study has a limited sample size which may affect the generalizability of the findings. Studies with a larger sample size may increase the validity of the results for larger populations. Second, some researchers currently use the “C1” and “C2” type classification instead of the “C” tympanogram classification. Different classifications may lead to different results in studies. Therefore, it is recommended that future studies be conducted with a larger dataset and considering different tympanogram classifications. Studies in the literature are mostly aimed at the diagnosis of otitis media with effusion. More studies on WBA with larger sample sizes are needed in the follow-up of otitis media with effusion.

## Conclusions

Our findings indicate that WBA is more successful than 226 Hz tympanometry in detecting small changes in the follow-up of otitis media with effusion. In this context, it is recommended that WBA findings be included in the follow-up of otitis media with effusion.
